# Silver(I) nitrate complexes of three tetra­kis-thio­ether-substituted pyrazine ligands: metal–organic chain, network and framework structures

**DOI:** 10.1107/S2056989017002791

**Published:** 2017-02-24

**Authors:** Tokouré Assoumatine, Helen Stoeckli-Evans

**Affiliations:** a CanAm Bioresearch Inc., 9-1250 Waverley Street, Winnipeg, Manitoba R3T 6C6, Canada; b Institute of Physics, University of Neuchâtel, rue Emile-Argand11, CH-2000 Neuchâtel, Switzerland

**Keywords:** crystal structure, tetra­kis-thio­ether-substituted pyrazines, silver(I) nitrate, metal–organic chain (MOC), metal–organic network (MON), metal–organic framework (MOF), C—H⋯O and C—H⋯S hydrogen bonds

## Abstract

The reaction of the ligand 2,3,5,6-tetra­kis­[(methyl­sulfanyl)­meth­yl]pyrazine (**L1**) with silver(I) nitrate led to {[Ag(C_12_H_20_N_2_S_4_)](NO_3_)}_*n*_, (I), *catena*-poly[[silver(I)-*μ*-2,3,5,6-tetra­kis­[(methyl­sulfan­yl)meth­yl]pyrazine] nitrate], a compound with a metal–organic chain structure. The asymmetric unit is composed of two half ligands, located about inversion centres, with one ligand coordinating to the silver atoms in a bis-tridentate manner and the other in a bis-bidentate manner. The charge on the metal atom is compensated for by a free nitrate anion. Hence, the silver atom has a fivefold S_3_N_2_ coordination sphere. The reaction of the ligand 2,3,5,6-tetra­kis­[(phenyl­sulfanyl)­meth­yl]pyrazine (**L2**) with silver(I) nitrate, led to [Ag_2_(NO_3_)_2_(C_32_H_28_N_2_S_4_)]_*n*_, (II), poly[di-*μ*-nitrato-bis­{*μ*-2,3,5,6-tetra­kis­[(phenyl­sulfan­yl)meth­yl]pyrazine}disilver], a compound with a metal–organic network structure. The asymmetric unit is composed of half a ligand, located about an inversion centre, that coordinates to the silver atoms in a bis-tridentate manner. The nitrate anion coordinates to the silver atom in a bidentate/monodentate manner, bridging the silver atoms, which therefore have a sixfold S_2_NO_3_ coordination sphere. The reaction of the ligand 2,3,5,6-tetra­kis­[(pyridin-2-yl­sulfanyl)­meth­yl]pyrazine (**L3**) with silver(I) nitrate led to [Ag_3_(NO_3_)_3_(C_28_H_24_N_6_S_4_)]_*n*_, (III), poly[trinitrato{*μ*_6_-2,3,5,6-tetra­kis[(pyri­din-2-ylsulfan­yl)meth­yl]pyrazine}­trisilver(I)], a compound with a metal–organic framework structure. The asymmetric unit is composed of half a ligand, located about an inversion centre, that coordinates to the silver atoms in a bis-tridentate manner. One pyridine N atom bridges the monomeric units, so forming a chain structure. Two nitrate O atoms also coordinate to this silver atom, hence it has a sixfold S_2_N_2_O_2_ coordination sphere. The chains are linked *via* a second silver atom, located on a twofold rotation axis, coordinated by the second pyridine N atom. A second nitrate anion, also lying about the twofold rotation axis, coordinates to this silver atom *via* an Ag—O bond, hence this second silver atom has a threefold N_2_O coordination sphere. In the crystal of (I), the nitrate anion plays an essential role in forming C—H⋯O hydrogen bonds that link the metal–organic chains to form a three-dimensional supra­molecular structure. In the crystal of (II), the metal–organic networks (lying parallel to the *bc* plane) stack up the *a-*axis direction but there are no significant inter­molecular inter­actions present between the layers. In the crystal of (III), there are a number of C—H⋯O hydrogen bonds present within the metal–organic framework. The role of the nitrate anion in the formation of the coordination polymers is also examined.

## Abstract 2

The reaction of silver nitrate with the ligands 2,3,5,6-tetra­kis­[(methyl­sulfanyl)­meth­yl]pyrazine, 2,3,5,6-tetra­kis­[(phenyl­sulfanyl)­meth­yl]pyrazine and 2,3,5,6-tetra­kis­[(pyridin-2-yl­sulfanyl)­meth­yl]pyrazine, three tetra­kis-thio­ether-substituted pyrazine ligands, lead, respectively, to the formation of compounds with a metal–organic chain, a metal–organic network and a metal–organic framework structure.

## Chemical context   

A series of tetra­kis-thio­ether pyrazine ligands have been prepared in order to study their coordination behaviour with various transition metals (Assoumatine, 1999 ▸). The ligands 2,3,5,6-tetra­kis­[(methyl­sulfanyl)­meth­yl]pyrazine (**L1**), 2,3,5,6-tetra­kis­[(phenyl­sulfanyl)­meth­yl]pyrazine (**L2**) and 2,3,5,6-tetra­kis­[(pyridin-2-yl­sulfanyl)­meth­yl]pyrazine (**L3**), were synthesized by the reaction of 2,3,5,6-tetra­kis­(bromo­meth­yl)pyrazine (Assoumatine & Stoeckli-Evans, 2014*b* ▸), with the appropriate 2-mercapto derivative. Their crystal structures and syntheses have been reported previously: **L1** (Assoumatine & Stoeckli-Evans, 2014*a* ▸), **L2** (Assoumatine *et al.*, 2007 ▸) and **L3** (Assoumatine & Stoeckli-Evans, 2016 ▸). The reaction of similar ligands with various silver(I) salts have also resulted in the formation of coordination polymers. For example, 2-{[(pyridin-4-ylmeth­yl)sulfanyl]­meth­yl}pyrazine (Black & Hanton, 2007 ▸) led to metal–organic frameworks, while ligands 2,3-bis­{[(pyridin-2-ylmeth­yl)sulfanyl]­meth­yl}pyrazine (Cara­doc-Davies & Hanton, 2001 ▸) and 2,5-bis­{[(pyridin-2-ylmeth­yl)sulfanyl]­meth­yl}pyrazine (Caradoc-Davies *et al.*, 2001 ▸) both resulted in compounds with metal–organic chains.
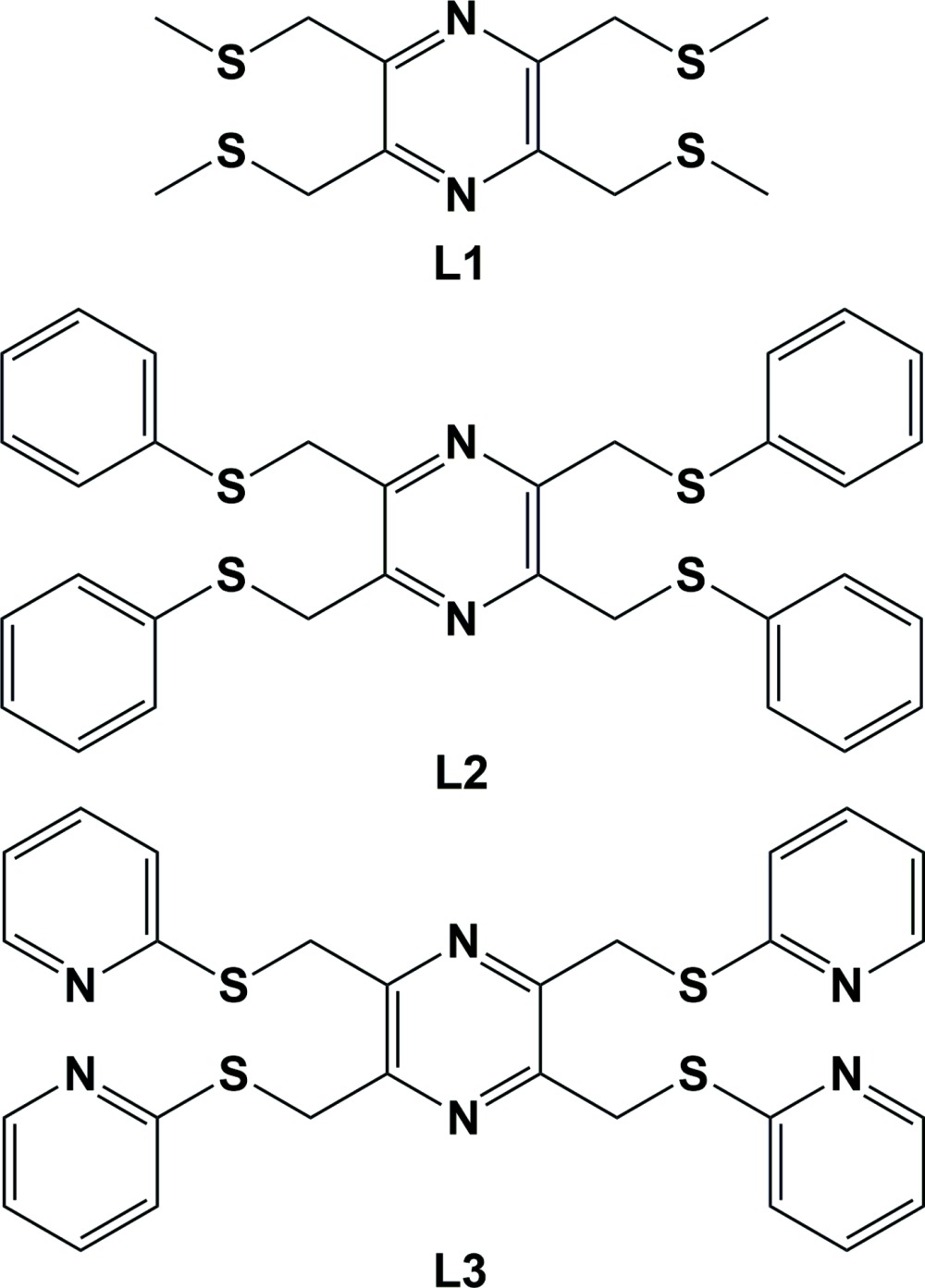
 
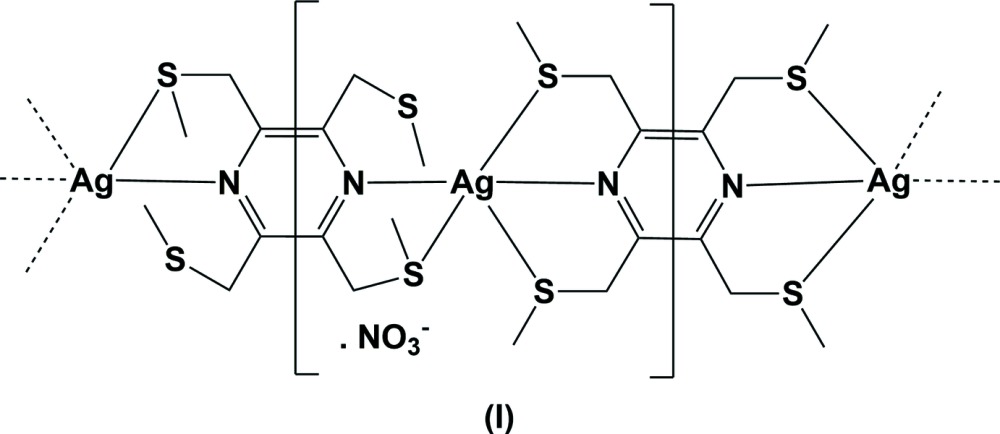
 
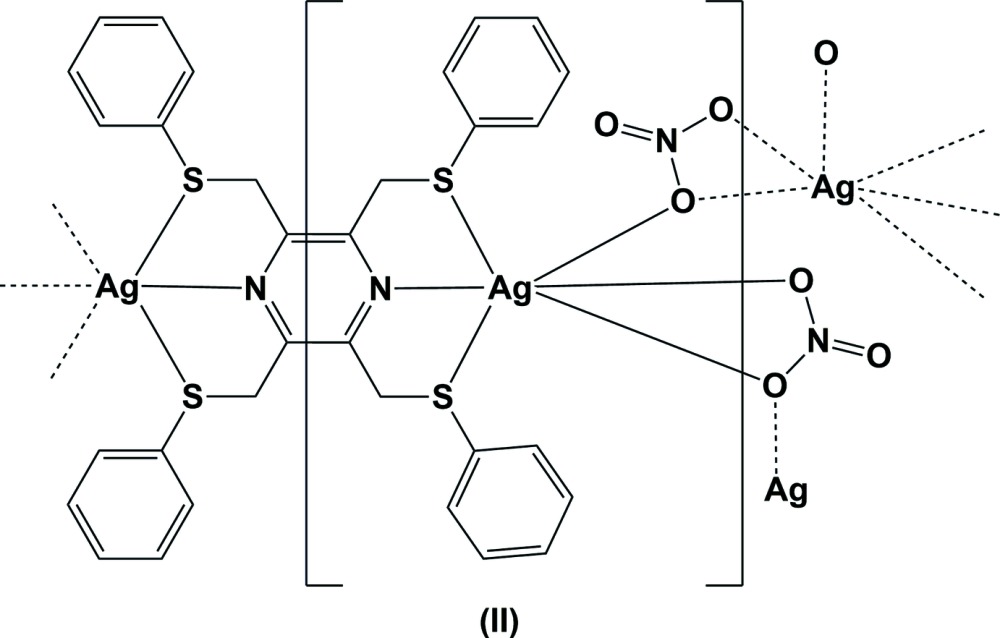
 
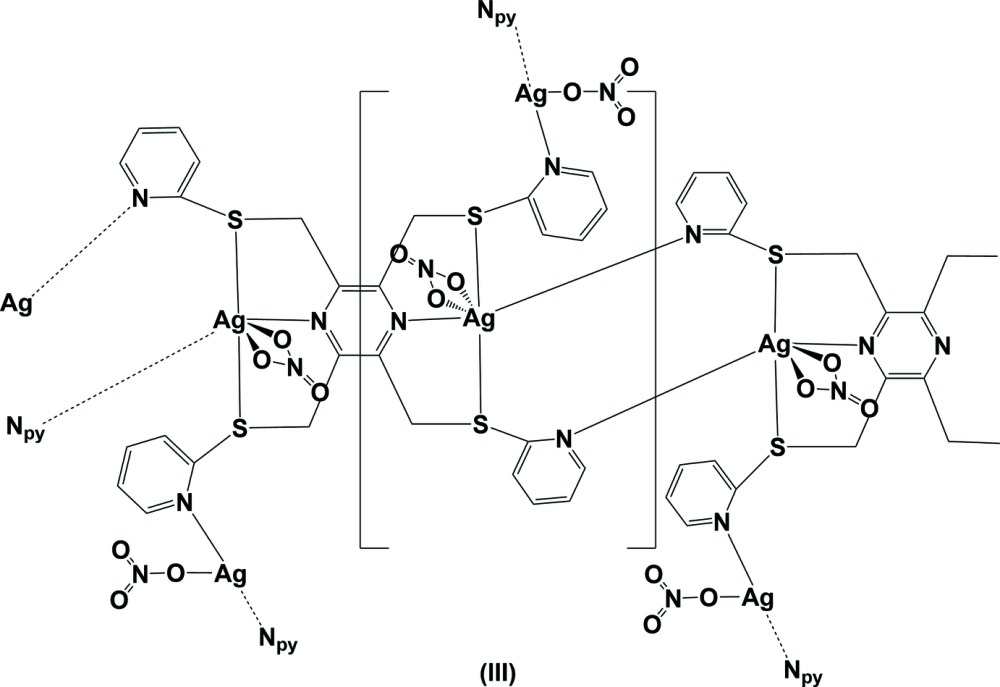


## Structural commentary   

**Figure 1:**
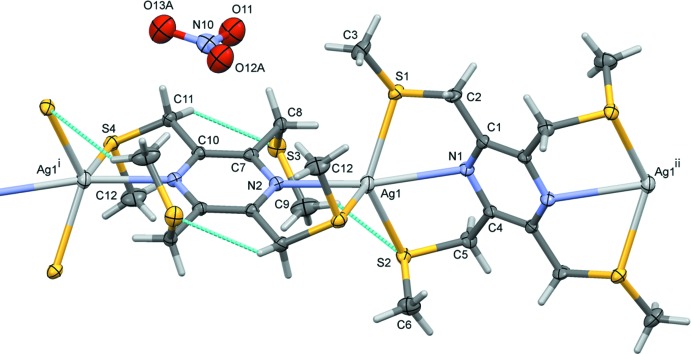
The mol­ecular entities of compound (I), with atom labelling for the asymmetric unit. Unlabelled atoms are related to labelled atoms by symmetry operation (i) = −*x*, −*y* + 1, −*z* + 1, for the ligand involving atom N2, and by symmetry operation (ii) = −*x* + 1, −*y* + 1, −*z* + 2, for the ligand involving atom N1. Displacement ellipsoids are drawn at the 50% probability level. The intra­molecular C—H⋯S contacts are shown as dashed lines (see Table 4 ▸).

The reaction of the ligand 2,3,5,6-tetra­kis­[(methyl­sulfanyl)­meth­yl]pyrazine (**L1**) with silver(I) nitrate, led to the formation of a metal–organic chain (MOC) structure, (I) (Fig. 1 ▸). Selected bond lengths and angles involving the Ag1 atom are given in Table 1 ▸. The asymmetric unit is composed of two half ligands, located about inversion centres, with one ligand coordinating to the silver atom in a bis-tridentate manner and the other in a bis-bidentate manner. Their pyrazine rings are almost normal to one another, making a dihedral angle of 88.6 (2)°. The charge on the metal atom is compensated for by a free nitrate anion. The silver atom, Ag1, has a fivefold S_3_N_2_ coordination sphere with a highly distorted shape and a *τ*_5_ value of 0.63 (*τ*_5_ = 0 for an ideal square-pyramidal coordination sphere, and = 1 for an ideal trigonal-pyramidal coordination sphere; Addison *et al.*, 1984 ▸). Within the MOC structure, there are significant C—H⋯S inter­actions present, involving the thio­ether substituent that does not coordinate to the silver atom, *viz.* atom S3 (Table 4 and Fig. 1 ▸).

**Figure 2:**
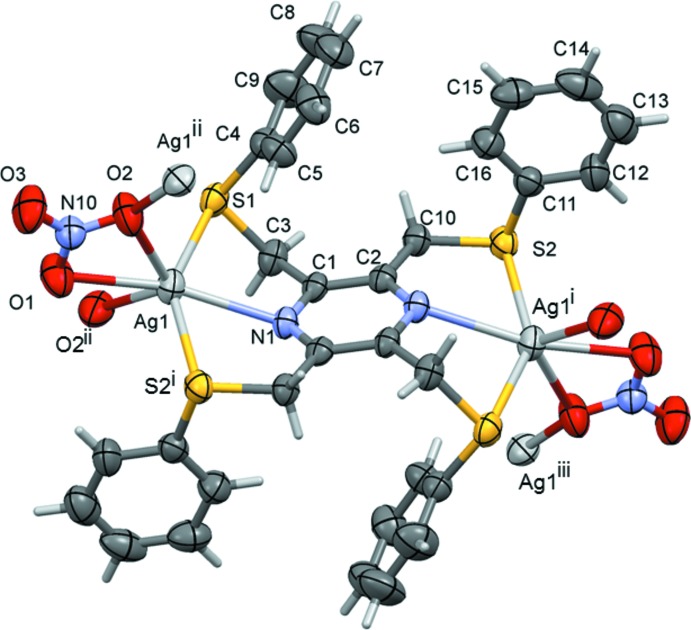
The mol­ecular entities of compound (II), with atom labelling for the asymmetric unit. For the ligand, unlabelled atoms are related to the labelled atoms by symmetry operation (i) −*x* + 2, −*y* + 2, −*z* + 1; other symmetry codes are (ii) *x*, −*y* + 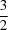, *z* + 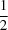; (iii) −*x* + 2, *y* + 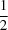, −*z* + 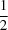. Displacement ellipsoids are drawn at the 50% probability level.

The reaction of the ligand 2,3,5,6-tetra­kis­[(phenyl­sulfanyl)­meth­yl]pyrazine (**L2**) with silver(I) nitrate, led to the formation of a metal–organic network (MON) structure, (II) (Fig. 2 ▸). Selected bond lengths and angles involving atom Ag1 are given in Table 2 ▸. The asymmetric unit is composed of half a ligand, located about an inversion centre, a silver atom and a nitrate anion. The ligand coordinates to the silver atoms in a bis-tridentate manner. The nitrate anion coordinates to the silver atom in a bidentate/monodentate manner, bridging the silver atoms, which therefore have a sixfold S_2_NO_3_ coordination sphere, best described as a highly distorted octa­hedron (Table 2 ▸).

**Figure 3:**
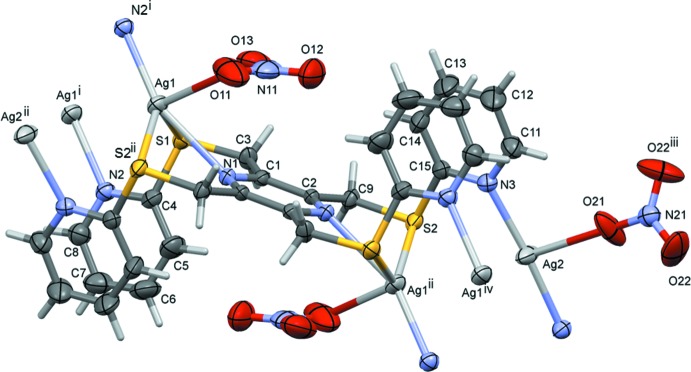
The mol­ecular entities of compound (III), with atom labelling for the asymmetric unit. For the ligand, unlabelled atoms are related to the labelled atoms by symmetry operation (ii) −*x* + 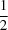, −*y* + 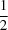, −*z*; other symmetry codes are (i) −*x*, −*y* + 1, −*z*; (iii) −*x* + 1, *y*, −*z* + 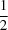; (iv) *x* + 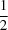, *y* − 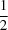, *z*. Displacement ellipsoids are drawn at the 50% probability level.

The reaction of the ligand 2,3,5,6-tetra­kis­[(pyridin-2-yl­sulfanyl)­meth­yl]pyrazine (**L3**) with silver(I) nitrate, led to the formation of a metal–organic framework (MOF) structure, (III) (Fig. 3 ▸). Selected bond lengths and angles involving atoms Ag1 and Ag2 are given in Table 3 ▸. The asymmetric unit is composed of half a ligand, located about an inversion centre, a silver atom and a nitrate anion, plus half a second AgNO_3_ unit located about a twofold rotation axis. The organic ligand coordinates to the silver atoms (Ag1), in a bis-tridentate manner. One pyridine N atom, N2, bridges the monomeric units, so forming a chain structure along the *b-*axis direction. The nitrate O atoms, O11 and O13, coordinate to silver atom Ag1, hence it has a highly distorted octa­hedral S_2_N_2_O_2_ coord­ination sphere (Table 3 ▸). The chains are linked *via* a second silver atom, Ag2, located on a twofold rotation axis, coordinated by the second pyridine N atom, N3. A second nitrate anion, also lying about the twofold rotation axis, coordinates to this silver atom *via* an Ag2—O21 bond, hence silver atom Ag2 has a T-shaped N_2_O coordination sphere.

It can be seen from Tables 1 ▸–3 ▸ ▸ that the Ag—N(pyrazine) and Ag—S bond lengths differ considerably for the three compounds. In compound (I), the Ag1—N2 bond length, involving the ligand that coordinates in a bis-bidentate manner, is considerably shorter at 2.436 (5) Å, compared to the Ag1—N1 bond length of 2.714 (4) Å, involving the ligand that coordinates in a bis-tridentate manner. These Ag—N(pyrazine) bond lengths contrast with those for compounds (II) and (III), where both ligands coordinate in a bis-tridentate manner, with values of 2.527 (4) and 2.578 (3) Å, respectively. The Ag1—S bond lengths in compound (I) are almost the same, varying from 2.5895 (15) to 2.5987 (16) Å. These distances are shorter than those in (II), which are 2.6560 (15) and 2.6790 (14) Å, but similar to bond length Ag1—S2^ii^ = 2.6010 (11) Å in (III). The longest Ag—S distance [2.7943 (13) Å] is found for bond Ag1—S1 in (III). Finally, in compound (III), the two Ag—N(pyridine) bond lengths also differ; Ag1—N2^i^ is 2.267 (3) Å, while bond length Ag2—N3 is shorter at 2.208 (3) Å (see Table 3 ▸). Despite the large variation in the Ag—N(pyrazine), Ag—S or Ag—N(pyridine) bond lengths, which perhaps indicates how flexible the ligands are, the values are within the limits observed for similar silver coordinating pyrazine, thio­ether or pyridine ligands, when compared to the values observed for such structures present in the Cambridge Structural Database (Groom *et al.*, 2016 ▸). The various histograms of the bond lengths have skewed-right distributions and the values vary from 2.10 to 2.75 Å for Ag—N(pyrazine), from 2.48 to 2.79 Å for Ag—S, and 1.90 to 2.99 Å for Ag—N(pyridine).

## Supra­molecular features   

**Figure 4:**
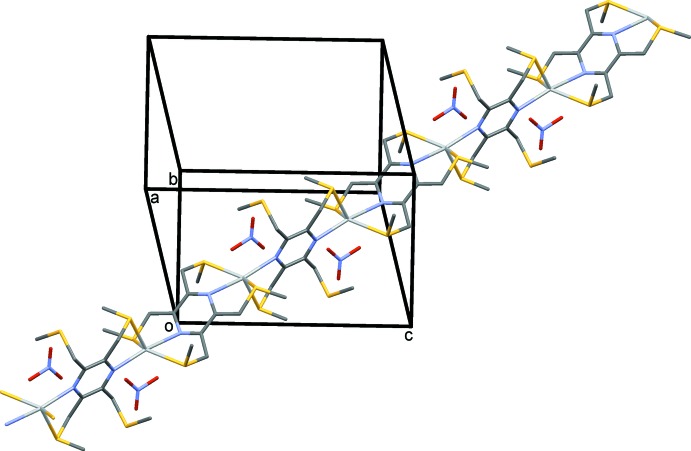
A partial view, normal to plane (1
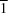
0), of the metal–organic chain structure of compound (I). The H atoms have been omitted for clarity

**Figure 5:**
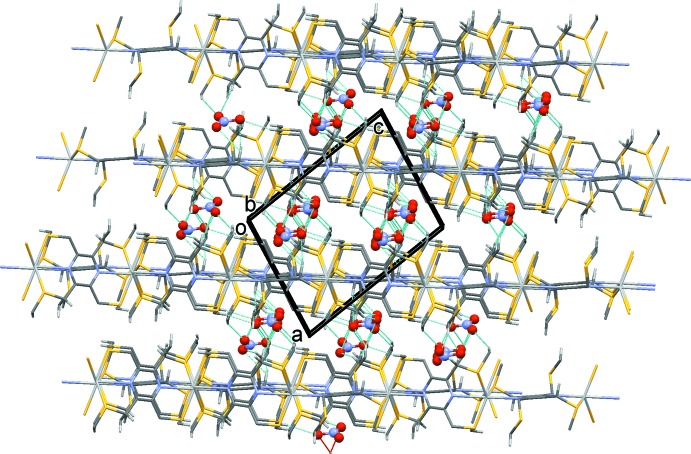
A view along the *b* axis of compound (I), with emphasis on the crystal packing. Hydrogen bonds are shown as dashed lines (see Table 4 ▸), and only those H atoms involved in inter­molecular C—H⋯O hydrogen bonds have been included.

In the crystal of (I), the metal–organic chains (Fig. 4 ▸) propagate along [101]. They are linked *via* a number of C—H⋯O hydrogen bonds (Table 4 ▸), forming a three-dimensional supra­molecular structure, as illustrated in Fig. 5 ▸.

**Figure 6:**
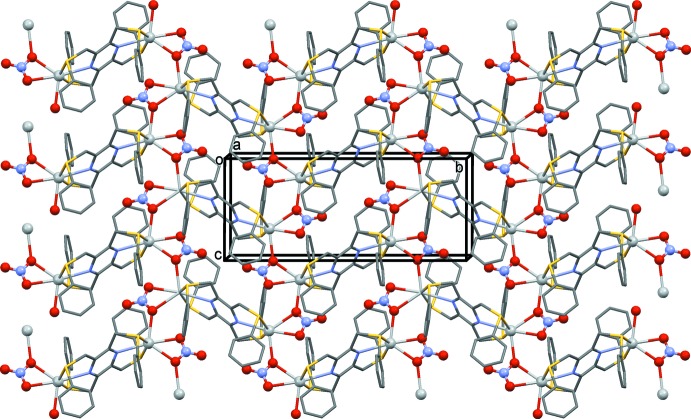
A view along the *a* axis of compound (II), illustrating the role of the NO_3_^−^ anion in forming the network structure. H atoms have been omitted for clarity

In the crystal of (II), the metal–organic networks extend parallel to the *bc* plane and stack up the *a* axis (Fig. 6 ▸), but there are no significant inter­molecular inter­actions present between the layers.

**Figure 7:**
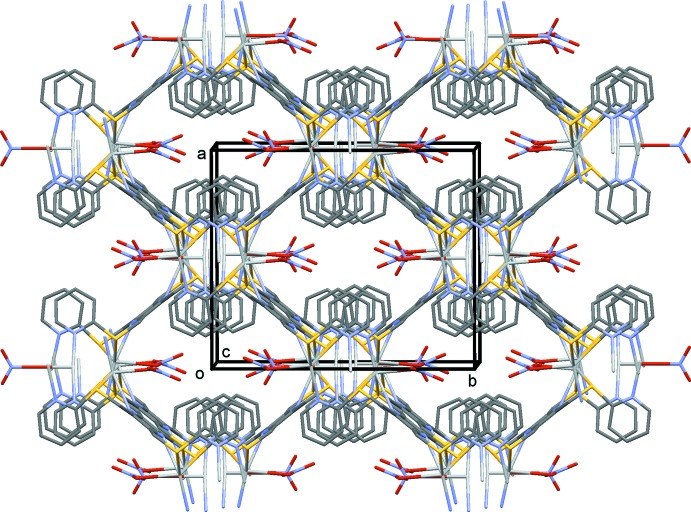
A view along the *c* axis of compound (III). H atoms have been omitted for clarity

In the crystal of (III), the metal–organic framework (Fig. 7 ▸) is reinforced by a number of C—H⋯O hydrogen bonds (Table 5 ▸). The voids in this three-dimensional structure, occupied by disordered solvent mol­ecules, amount to only *ca* 3.7% of the total volume of the unit cell.

## Database survey   

A search of the Cambridge Structural Database (Version 5.38, first update November 2016; Groom *et al.*, 2016 ▸) for tetra­kis-substituted pyrazine ligands gave 774 hits, which include 194 hits for compounds involving tetra­methyl­pyrazine. The first such ligand, tetra­kis-2,3,5,6-(2′-pyrid­yl)pyrazine, was synthesized by Goodwin & Lions (1959 ▸), and the crystal structures of three polymorphs have been reported; a monoclininc *P*2_1_/*n* polymorph (VUKGAJ01; Bock *et al.*, 1992 ▸), a tetra­gonal *I*4_1_/*a* polymorph (VUKGAJ; Greaves & Stoeckli-Evans, 1992 ▸) and a second monoclinic *C*2/*c* polymorph (VUKGAJ03; Behrens & Rehder, 2009 ▸). The most recent tetra­kis-substituted pyrazine ligand to be described is *N*,*N*′,*N*′′,*N*′′′-tetra­ethyl­pyrazine-2,3,5,6-tetra­carboxamide (OSUTIH; Lohrman *et al.*, 2016 ▸). In the last update of the CSD there are a total of three tetra­kis-substituted thio­ether pyrazine compounds, *viz*. two polymorphs of compound 2,3,5,6-tetra­kis­(naphthalen-2-ylsulf­an­yl­meth­yl)pyrazine (Pacifico & Stoeckli-Evans, 2004 ▸), and the ligands **L1** and **L2**.

The role of the anion in coordination chemistry is often essential for the formation of multi-dimensional structures. The nitrate anion can be present as an isolated anion, coordinating to the metal atom or even bridging metal atoms. A search of the CSD for silver nitrate complexes yielded 2192 hits, among which it was noted that the nitrate anion can coordinate in at least 10 different manners. In the present study, three different situations are observed. In (I), the nitrate anion is present as an isolated anion. Its role here is to form C—H⋯O hydrogen bonds, resulting in the formation of a three-dimensional supra­molecular structure (Fig. 5 ▸ and Table 4 ▸). In (II), the nitrate anion is essential in forming the network structure. The **–Ag–L2–Ag–L2–** chains, which propagate along [010], are linked by the nitrate anion in the [001] direction, so forming the metal–organic network (Fig. 6 ▸ and Table 2 ▸). Finally, there are two independent nitrate anions present in (III). They coordinate to the metal atoms in different manners, but they do not appear to be the essential elements in forming the three-dimensional framework (Fig. 7 ▸ and Table 3 ▸). Here, it is the presence of the pyridine rings, which twist about the S—C_ar_ bonds, that enables the metal atoms to cross-link, so forming the metal–organic framework.

## Synthesis and crystallization   

**Compound (I):**

A solution of **L1** (50 mg, 0.16 mmol; Assoumatine & Stoeckli-Evans, 2014*a* ▸) in CH_2_Cl_2_ (5 ml) was introduced into a 16 mm diameter glass tube and layered with MeCN (2 ml) as a buffer zone. Then a solution of AgNO_3_ (27 mg, 0.16 mmol) in MeCN (5 ml) was added very gently to avoid possible mixing. The glass tube was sealed and left in the dark at room temperature for at least two weeks, whereupon yellow plate-like crystals of complex (I) were isolated at the inter­face between the two solutions. IR (KBr disc, cm^−1^): ν = 2985 *w*, 2912 *w*, 1406 *bm*, 1341 *bs*, 1141 *w*, 1115 *w*, 982 *w*, 828 *w*, 777 *w*, 701 *vw*, 478 *vw*.

**Compound (II):**

A solution of **L2** (50 mg, 0.09 mmol; Assoumatine *et al.*, 2007 ▸) in THF (5 ml) was introduced into a 16 mm diameter glass tube and layered with MeCN (2 ml) as a buffer zone. Then a solution of AgNO_3_ (15 mg, 0.09 mmol) in MeCN (5 ml) was added very gently to avoid possible mixing. The glass tube was sealed and left in the dark at room temperature for at least three weeks, whereupon yellow block-like crystals of complex (II) were isolated from the bottom of the tube. IR (KBr disc, cm^−1^): ν = 3053 *vw*, 2962 *vw*, 2927 *vw*, 1583 *w*, 1480 *w*, 1386 *bs*, 1278 *vs*, 1133 *vw*, 1023 *w*, 850 *vw*, 738 *s*, 690 *m*, 495 *vw*, 478 *vw*.

**Compound (III):**

A solution of **L3** (50 mg, 0.09 mmol; Assoumatine & Stoeckli-Evans, 2016 ▸) in CHCl_3_ (5 ml) was introduced into a 16 mm diameter glass tube and layered with MeCN (2 ml) as a buffer zone. Then a solution of AgNO_3_ (15 mg, 0.09 mmol) in MeCN (5 ml) was added very gently to avoid possible mixing. The glass tube was sealed and left in the dark at room temperature for at least two weeks, whereupon pale-yellow needle-like crystals of complex (III) were isolated at the inter­face between the two solutions. IR (KBr disc, cm^−1^): ν = 3097 *vw*, 2899 *vw*, 1581 *m*, 1562 *w*, 1460 *m*, 1386 *bs*, 1305 *bs*, 1163 *w*, 1126 *w*, 1032 *vw*, 1004 *vw*, 825 *vw*, 759 *m*, 723 *vw*, 461*vw*.

## Refinement   

Crystal data, data collection and structure refinement details are summarized in Table 6 ▸. Complexes (I) and (II) were measured at 293 K on a four-circle diffractometer, while complex (III) was measured at 223 K on a one-circle image-plate diffractometer. In complex (I), the nitrate ion is positionally disordered and atoms O12*A/*O12*B* and O13*A*/O13*B* were refined with a fixed occupancy ratio of 0.5:0.5. No absorption correction was applied for complex (II) owing to the irregular shape of the crystal, and as there were no suitable reflections for *ψ* scans. For complex (III), a region of disordered electron density (25 electrons for a solvent-accessible volume of 130 Å^3^) was corrected for using the SQUEEZE routine in *PLATON* (Spek, 2015 ▸). Their formula mass and unit-cell characteristics were not taken into account for the final model. For complexes (I) and (II), only one equivalent of data were measured, hence *R*_int_ = 0. In all three complexes, the H atoms were included in calculated positions and refined as riding: C—H = 0.96–0.97 Å for (I), 0.93–0.97 Å for (II) and 0.94–0.98 Å for (III), with *U*_iso_(H) = 1.5*U*_eq_(C-meth­yl) and 1.2*U*_eq_(C) for other H atoms.

## Supplementary Material

Crystal structure: contains datablock(s) I, II, III, Global. DOI: 10.1107/S2056989017002791/wm5369sup1.cif

Structure factors: contains datablock(s) I. DOI: 10.1107/S2056989017002791/wm5369Isup2.hkl

Structure factors: contains datablock(s) II. DOI: 10.1107/S2056989017002791/wm5369IIsup3.hkl

Structure factors: contains datablock(s) III. DOI: 10.1107/S2056989017002791/wm5369IIIsup4.hkl

CCDC references: 1533573, 1533572, 1533571

Additional supporting information:  crystallographic information; 3D view; checkCIF report

## Figures and Tables

**Table 1:** Selected geometric parameters (Å, °) for (I)

Ag1—N1	2.714 (4)	Ag1—S2	2.5987 (16)
Ag1—N2	2.436 (5)	Ag1—S4^i^	2.5910 (15)
Ag1—S1	2.5895 (15)		
			
N1—Ag1—N2	167.75 (13)	N2—Ag1—S2	109.60 (11)
N1—Ag1—S1	64.36 (9)	N2—Ag1—S4^i^	77.43 (10)
N1—Ag1—S2	72.54 (9)	S1—Ag1—S2	129.99 (5)
N1—Ag1—S4^i^	113.79 (9)	S1—Ag1—S4^i^	111.41 (5)
N2—Ag1—S1	107.74 (11)	S4^i^—Ag1—S2	108.26 (5)

Symmetry code: (i) 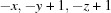.

**Table 2:** Selected geometric parameters (Å, °) for (II)

Ag1—N1	2.527 (4)	Ag1—O1	2.551 (4)
Ag1—S1	2.6560 (15)	Ag1—O2	2.507 (4)
Ag1—S2^i^	2.6790 (14)	Ag1—O2^ii^	2.539 (4)
			
N1—Ag1—S1	76.40 (9)	O2^ii^—Ag1—O1	49.56 (12)
N1—Ag1—S2^i^	70.89 (9)	O2—Ag1—S1	80.10 (11)
S1—Ag1—S2^i^	146.98 (4)	O2^ii^—Ag1—S1	101.67 (11)
O2—Ag1—N1	112.54 (12)	O1—Ag1—S1	120.09 (11)
O2—Ag1—O2^ii^	117.32 (8)	O2—Ag1—S2^i^	116.46 (10)
N1—Ag1—O2^ii^	128.98 (12)	O2^ii^—Ag1—S2^i^	95.47 (11)
O2—Ag1—O1	75.15 (13)	O1—Ag1—S2^i^	92.47 (11)
N1—Ag1—O1	163.34 (14)		

Symmetry codes: (i) 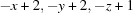; (ii) 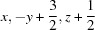.

**Table 3:** Selected geometric parameters (Å, °) for (III)

Ag1—N1	2.578 (3)	Ag1—O11	2.700 (5)
Ag1—N2^i^	2.267 (3)	Ag1—O13	2.752 (5)
Ag1—S1	2.7943 (13)	Ag2—N3	2.208 (3)
Ag1—S2^ii^	2.6010 (11)	Ag2—O21	2.567 (5)
			
N1—Ag1—N2^i^	155.31 (11)	S2^ii^—Ag1—O13	120.26 (10)
S1—Ag1—S2^ii^	122.71 (3)	O11—Ag1—N1	73.76 (11)
S1—Ag1—N1	68.98 (7)	O11—Ag1—N2^i^	99.33 (12)
S1—Ag1—N2^i^	96.92 (8)	O13—Ag1—N1	69.73 (11)
S2^ii^—Ag1—N1	70.29 (7)	O13—Ag1—N2^i^	88.28 (12)
S2^ii^—Ag1—N2^i^	133.03 (8)	O11—Ag1—O13	45.99 (14)
S1—Ag1—O11	122.18 (10)	N3—Ag2—N3^iii^	175.41 (12)
S1—Ag1—O13	79.78 (10)	O21—Ag2—N3	92.30 (9)
S2^ii^—Ag1—O11	81.18 (10)		

Symmetry codes: (i) 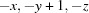; (ii) 
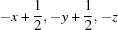
; (iii) 
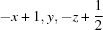
.

**Table 4:** Hydrogen-bond geometry (Å, °) for (I)

*D*—H⋯*A*	*D*—H	H⋯*A*	*D*⋯*A*	*D*—H⋯*A*
C9—H9*C*⋯S2	0.96	2.86	3.650 (8)	141
C11—H11*A*⋯S3	0.97	2.74	3.502 (6)	136
C11—H11*B*⋯O13*A*	0.97	2.52	3.438 (17)	157
C2—H2*A*⋯O11^ii^	0.97	2.55	3.460 (9)	156
C2—H2*B*⋯O12*A* ^iii^	0.97	2.53	3.431 (15)	154
C3—H3*C*⋯O12*A* ^iii^	0.96	2.37	3.171 (17)	141
C3—H3*C*⋯O12*B* ^iii^	0.96	2.57	3.364 (16)	140
C6—H6*A*⋯O13*A* ^i^	0.96	2.52	3.375 (19)	149
C9—H9*A*⋯O11^iv^	0.96	2.58	3.503 (10)	162

Symmetry codes: (i) 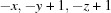; (ii) 
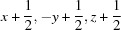
; (iii) 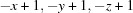; (iv) 
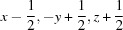
.

**Table 5:** Hydrogen-bond geometry (Å, °) for (III)

*D*—H⋯*A*	*D*—H	H⋯*A*	*D*⋯*A*	*D*—H⋯*A*
C11—H11⋯O21	0.94	2.57	3.287 (5)	133
C3—H3*B*⋯O21^iv^	0.98	2.40	3.253 (4)	145
C3—H3*B*⋯O22^iv^	0.98	2.49	3.420 (6)	158
C7—H7⋯O13^v^	0.94	2.51	3.268 (6)	138
C9—H9*A*⋯O22^iv^	0.98	2.32	3.291 (6)	171
C12—H12⋯O11^vi^	0.94	2.51	3.310 (7)	142
C14—H14⋯O22^iv^	0.94	2.59	3.349 (7)	138

Symmetry codes: (iv) 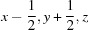; (v) 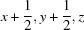; (vi) 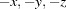.

**Table 6:** Experimental details

	(I)	(II)	(III)
Crystal data			
Chemical formula	[Ag(C_12_H_20_N_2_S_4_](NO_3_)	[Ag_2_(NO_3_)_2_(C_32_H_28_N_2_S_4_)]	[Ag_3_(NO_3_)_3_(C_28_H_24_N_6_S_4_)]
*M*_r_	490.42	908.56	1082.41
Crystal system, space group	Monoclinic, *P*2_1_/*n*	Monoclinic, *P*2_1_/*c*	Monoclinic, *C*2/*c*
Temperature (K)	293	293	223
*a*, *b*, *c* (Å)	10.167 (2), 13.482 (3), 13.377 (3)	11.8437 (14), 18.5674 (14), 7.8444 (12)	13.6319 (9), 16.2211 (10), 15.7201 (11)
β (°)	100.838 (19)	96.856 (11)	96.607 (8)
*V* (Å^3^)	1800.9 (7)	1712.7 (4)	3453.0 (4)
*Z*	4	2	4
Radiation type	Mo *K*α	Mo *K*α	Mo *K*α
μ (mm^−1^)	1.60	1.44	1.99
Crystal size (mm)	0.61 × 0.61 × 0.17	0.46 × 0.46 × 0.38	0.45 × 0.08 × 0.08

Data collection			
Diffractometer	Stoe AED2 4-circle	Stoe AED2 4-circle	STOE IPDS1
Absorption correction	Analytical (*ABST*; Spek, 2009 ▸)	–	Multi-scan (*MULABS*; Spek, 2009 ▸)
*T*_min_, *T*_max_	0.457, 0.789	–	0.949, 1.000
No. of measured, independent and observed [*I* > 2σ(*I*)] reflections	3318, 3318, 2857	3178, 3178, 2606	13264, 3311, 1936
*R*_int_	0	0	0.072
(sin θ/λ)_max_ (Å^−1^)	0.607	0.606	0.614

Refinement			
*R*[*F* ^2^ > 2σ(*F* ^2^)], *wR*(*F* ^2^), *S*	0.056, 0.161, 1.09	0.045, 0.100, 1.16	0.030, 0.052, 0.76
No. of reflections	3318	3178	3311
No. of parameters	207	218	242
H-atom treatment	H-atom parameters constrained	H-atom parameters constrained	H-atom parameters constrained
Δρ_max_, Δρ_min_ (e Å^−3^)	1.97, −1.50	0.62, −0.61	0.43, −0.44

Computer programs: *STADI4* and *X-RED* (Stoe & Cie, 1997 ▸), *EXPOSE*, *CELL* and *INTEGRATE* in *IPDS-I* (Stoe & Cie, 1998 ▸), *SHELXS97* (Sheldrick, 2008 ▸), *SHELXL2014/6* (Sheldrick, 2015 ▸), *Mercury* (Macrae *et al.*, 2008 ▸), *PLATON* (Spek, 2009 ▸) and *publCIF* (Westrip, 2010 ▸).

## supplementary crystallographic information

### (I) catena-Poly[[silver(I)-µ-2,3,5,6-tetrakis[(methylsulfanyl)methyl]pyrazine] nitrate] Crystal data

**Table:**

[Ag(C_12_H_20_N_2_S_4_](NO_3_)	*F*(000) = 992
*M**_r_* = 490.42	*D*_x_ = 1.809 Mg m^−^^3^
Monoclinic, *P*2_1_/*n*	Mo *K*α radiation, λ = 0.71073 Å
*a* = 10.167 (2) Å	Cell parameters from 31 reflections
*b* = 13.482 (3) Å	θ = 14.1–19.7°
*c* = 13.377 (3) Å	µ = 1.60 mm^−^^1^
β = 100.838 (19)°	*T* = 293 K
*V* = 1800.9 (7) Å^3^	Plate, yellow
*Z* = 4	0.61 × 0.61 × 0.17 mm

### (I) catena-Poly[[silver(I)-µ-2,3,5,6-tetrakis[(methylsulfanyl)methyl]pyrazine] nitrate] Data collection

**Table:**

Stoe AED2 4-circle diffractometer	2857 reflections with *I* > 2σ(*I*)
Radiation source: fine-focus sealed tube	*R*_int_ = 0.000
Graphite monochromator	θ_max_ = 25.6°, θ_min_ = 2.2°
ω/2θ scans	*h* = −11→12
Absorption correction: analytical (*ABST*; Spek, 2009)	*k* = 0→16
*T*_min_ = 0.457, *T*_max_ = 0.789	*l* = 0→16
3318 measured reflections	2 standard reflections every 120 min
3318 independent reflections	intensity decay: 5%

### (I) catena-Poly[[silver(I)-µ-2,3,5,6-tetrakis[(methylsulfanyl)methyl]pyrazine] nitrate] Refinement

**Table:**

Refinement on *F*^2^	Secondary atom site location: difference Fourier map
Least-squares matrix: full	Hydrogen site location: inferred from neighbouring sites
*R*[*F*^2^ > 2σ(*F*^2^)] = 0.056	H-atom parameters constrained
*wR*(*F*^2^) = 0.161	*w* = 1/[σ^2^(*F*_o_^2^) + (0.0974*P*)^2^ + 4.9525*P*] where *P* = (*F*_o_^2^ + 2*F*_c_^2^)/3
*S* = 1.09	(Δ/σ)_max_ < 0.001
3318 reflections	Δρ_max_ = 1.97 e Å^−^^3^
207 parameters	Δρ_min_ = −1.50 e Å^−^^3^
0 restraints	Extinction correction: SHELXL-2016/6 (Sheldrick 2015), Fc^*^=kFc[1+0.001xFc^2^λ^3^/sin(2θ)]^-1/4^
Primary atom site location: structure-invariant direct methods	Extinction coefficient: 0.0035 (8)

### (I) catena-Poly[[silver(I)-µ-2,3,5,6-tetrakis[(methylsulfanyl)methyl]pyrazine] nitrate] Special details

**Table:**

Geometry. All esds (except the esd in the dihedral angle between two l.s. planes) are estimated using the full covariance matrix. The cell esds are taken into account individually in the estimation of esds in distances, angles and torsion angles; correlations between esds in cell parameters are only used when they are defined by crystal symmetry. An approximate (isotropic) treatment of cell esds is used for estimating esds involving l.s. planes.

### (I) catena-Poly[[silver(I)-µ-2,3,5,6-tetrakis[(methylsulfanyl)methyl]pyrazine] nitrate] Fractional atomic coordinates and isotropic or equivalent isotropic displacement parameters (Å^2^)

**Table:**

	*x*	*y*	*z*	*U*_iso_*/*U*_eq_	Occ. (<1)
Ag1	0.23796 (5)	0.48987 (4)	0.75616 (3)	0.0401 (2)	
S1	0.43682 (14)	0.37806 (10)	0.73224 (10)	0.0323 (3)	
S2	0.11067 (14)	0.50053 (10)	0.90707 (11)	0.0320 (3)	
S3	−0.06264 (16)	0.24651 (10)	0.63652 (12)	0.0413 (4)	
S4	−0.26369 (13)	0.33208 (10)	0.31050 (10)	0.0330 (3)	
N1	0.4152 (4)	0.4454 (3)	0.9286 (3)	0.0292 (9)	
N2	0.0779 (5)	0.4916 (3)	0.5959 (4)	0.0282 (10)	
C1	0.5407 (5)	0.4649 (4)	0.9166 (4)	0.0272 (11)	
C2	0.5796 (6)	0.4261 (5)	0.8198 (4)	0.0360 (13)	
H2A	0.645719	0.373887	0.836842	0.043*	
H2B	0.620036	0.479211	0.787138	0.043*	
C3	0.4998 (7)	0.3836 (5)	0.6154 (5)	0.0486 (16)	
H3A	0.430481	0.364140	0.559808	0.073*	
H3B	0.574665	0.339421	0.619518	0.073*	
H3C	0.527752	0.450091	0.604654	0.073*	
C4	0.3736 (5)	0.4814 (4)	1.0107 (4)	0.0268 (11)	
C5	0.2294 (5)	0.4566 (4)	1.0169 (4)	0.0322 (11)	
H5A	0.207925	0.486153	1.078010	0.039*	
H5B	0.220455	0.385218	1.022356	0.039*	
C6	0.1099 (7)	0.6312 (5)	0.9332 (6)	0.0512 (16)	
H6A	0.047093	0.663787	0.880930	0.077*	
H6B	0.197800	0.657895	0.934761	0.077*	
H6C	0.084358	0.641823	0.997925	0.077*	
C7	0.0317 (5)	0.4104 (4)	0.5426 (4)	0.0273 (10)	
C10	−0.0478 (5)	0.4186 (4)	0.4465 (4)	0.0265 (10)	
C11	−0.0956 (5)	0.3274 (4)	0.3842 (4)	0.0334 (12)	
H11A	−0.089767	0.271140	0.430044	0.040*	
H11B	−0.034496	0.315052	0.337994	0.040*	
C8	0.0713 (5)	0.3120 (4)	0.5925 (4)	0.0313 (11)	
H8A	0.143563	0.322787	0.650060	0.038*	
H8B	0.105664	0.270039	0.544339	0.038*	
C9	−0.0972 (9)	0.3316 (5)	0.7311 (6)	0.062 (2)	
H9A	−0.161874	0.302866	0.766452	0.093*	
H9B	−0.132267	0.392150	0.698934	0.093*	
H9C	−0.016152	0.345284	0.778694	0.093*	
C12	−0.3592 (7)	0.3497 (6)	0.4101 (6)	0.0569 (18)	
H12A	−0.451820	0.359680	0.380318	0.085*	
H12B	−0.326070	0.406797	0.449937	0.085*	
H12C	−0.350633	0.292137	0.453042	0.085*	
N10	0.2281 (6)	0.3702 (5)	0.3423 (5)	0.0544 (15)	
O11	0.2331 (7)	0.2979 (5)	0.3953 (5)	0.0842 (13)	
O12A	0.2834 (16)	0.4447 (11)	0.3565 (11)	0.0842 (13)	0.5
O13A	0.1631 (17)	0.3451 (10)	0.2526 (12)	0.0842 (13)	0.5
O12B	0.2588 (16)	0.4507 (11)	0.4035 (11)	0.0842 (13)	0.5
O13B	0.1847 (18)	0.3871 (10)	0.2537 (12)	0.0842 (13)	0.5

### (I) catena-Poly[[silver(I)-µ-2,3,5,6-tetrakis[(methylsulfanyl)methyl]pyrazine] nitrate] Atomic displacement parameters (Å^2^)

**Table:**

	*U*^11^	*U*^22^	*U*^33^	*U*^12^	*U*^13^	*U*^23^
Ag1	0.0397 (3)	0.0501 (3)	0.0284 (3)	0.00285 (18)	0.0014 (2)	0.00409 (17)
S1	0.0349 (7)	0.0359 (7)	0.0280 (7)	−0.0023 (5)	0.0105 (5)	−0.0025 (5)
S2	0.0243 (7)	0.0424 (8)	0.0293 (7)	−0.0031 (5)	0.0056 (5)	−0.0003 (5)
S3	0.0473 (9)	0.0327 (7)	0.0422 (8)	−0.0100 (6)	0.0044 (7)	0.0024 (6)
S4	0.0312 (7)	0.0361 (7)	0.0315 (7)	−0.0046 (5)	0.0056 (5)	−0.0069 (5)
N1	0.022 (2)	0.040 (2)	0.027 (2)	−0.0010 (18)	0.0078 (18)	−0.0002 (18)
N2	0.030 (2)	0.029 (2)	0.027 (2)	0.0020 (17)	0.0065 (19)	−0.0017 (16)
C1	0.028 (3)	0.035 (3)	0.020 (2)	0.005 (2)	0.007 (2)	0.0033 (19)
C2	0.029 (3)	0.054 (3)	0.029 (3)	0.002 (2)	0.016 (2)	−0.006 (2)
C3	0.061 (4)	0.056 (4)	0.031 (3)	−0.010 (3)	0.017 (3)	−0.011 (3)
C4	0.022 (3)	0.037 (3)	0.023 (2)	0.005 (2)	0.007 (2)	0.004 (2)
C5	0.023 (3)	0.041 (3)	0.034 (3)	−0.005 (2)	0.010 (2)	0.005 (2)
C6	0.060 (4)	0.038 (3)	0.059 (4)	0.003 (3)	0.022 (3)	0.004 (3)
C7	0.025 (2)	0.028 (2)	0.030 (3)	−0.0005 (19)	0.008 (2)	−0.002 (2)
C10	0.024 (2)	0.028 (2)	0.028 (2)	0.0012 (19)	0.007 (2)	−0.002 (2)
C11	0.031 (3)	0.033 (3)	0.034 (3)	0.000 (2)	0.001 (2)	−0.009 (2)
C8	0.034 (3)	0.031 (3)	0.027 (3)	0.001 (2)	0.000 (2)	0.003 (2)
C9	0.077 (5)	0.046 (4)	0.076 (5)	−0.009 (3)	0.047 (4)	−0.005 (4)
C12	0.056 (4)	0.062 (4)	0.063 (4)	−0.006 (3)	0.036 (4)	−0.003 (3)
N10	0.038 (3)	0.061 (4)	0.068 (4)	−0.006 (3)	0.020 (3)	0.006 (3)
O11	0.109 (4)	0.071 (3)	0.076 (3)	−0.016 (3)	0.026 (3)	0.011 (3)
O12A	0.109 (4)	0.071 (3)	0.076 (3)	−0.016 (3)	0.026 (3)	0.011 (3)
O13A	0.109 (4)	0.071 (3)	0.076 (3)	−0.016 (3)	0.026 (3)	0.011 (3)
O12B	0.109 (4)	0.071 (3)	0.076 (3)	−0.016 (3)	0.026 (3)	0.011 (3)
O13B	0.109 (4)	0.071 (3)	0.076 (3)	−0.016 (3)	0.026 (3)	0.011 (3)

### (I) catena-Poly[[silver(I)-µ-2,3,5,6-tetrakis[(methylsulfanyl)methyl]pyrazine] nitrate] Geometric parameters (Å, º)

**Table:**

Ag1—N1	2.714 (4)	C4—C5	1.522 (7)
Ag1—N2	2.436 (5)	C5—H5A	0.9700
Ag1—S1	2.5895 (15)	C5—H5B	0.9700
Ag1—S2	2.5987 (16)	C6—H6A	0.9600
Ag1—S4^i^	2.5910 (15)	C6—H6B	0.9600
S1—C3	1.798 (6)	C6—H6C	0.9600
S1—C2	1.805 (6)	C7—C10	1.389 (7)
S2—C6	1.797 (7)	C7—C8	1.506 (7)
S2—C5	1.817 (6)	C10—C11	1.513 (7)
S3—C9	1.791 (7)	C11—H11A	0.9700
S3—C8	1.812 (6)	C11—H11B	0.9700
S4—C11	1.806 (5)	C8—H8A	0.9700
S4—C12	1.806 (7)	C8—H8B	0.9700
N1—C4	1.341 (7)	C9—H9A	0.9600
N1—C1	1.342 (7)	C9—H9B	0.9600
N2—C7	1.342 (7)	C9—H9C	0.9600
N2—C10^i^	1.348 (6)	C12—H12A	0.9600
C1—C4^ii^	1.381 (8)	C12—H12B	0.9600
C1—C2	1.517 (7)	C12—H12C	0.9600
C2—H2A	0.9700	N10—O12A	1.148 (15)
C2—H2B	0.9700	N10—O11	1.202 (8)
C3—H3A	0.9600	N10—O13B	1.205 (17)
C3—H3B	0.9600	N10—O13A	1.301 (17)
C3—H3C	0.9600	N10—O12B	1.360 (16)
			
N1—Ag1—N2	167.75 (13)	C4—C5—H5B	109.1
N1—Ag1—S1	64.36 (9)	S2—C5—H5B	109.1
N1—Ag1—S2	72.54 (9)	H5A—C5—H5B	107.8
N1—Ag1—S4^i^	113.79 (9)	S2—C6—H6A	109.5
N2—Ag1—S1	107.74 (11)	S2—C6—H6B	109.5
N2—Ag1—S2	109.60 (11)	H6A—C6—H6B	109.5
N2—Ag1—S4^i^	77.43 (10)	S2—C6—H6C	109.5
S1—Ag1—S2	129.99 (5)	H6A—C6—H6C	109.5
S1—Ag1—S4^i^	111.41 (5)	H6B—C6—H6C	109.5
S4^i^—Ag1—S2	108.26 (5)	N2—C7—C10	120.8 (5)
C3—S1—C2	100.1 (3)	N2—C7—C8	116.5 (4)
C3—S1—Ag1	119.8 (2)	C10—C7—C8	122.8 (4)
C2—S1—Ag1	105.22 (19)	N2^i^—C10—C7	120.5 (4)
C6—S2—C5	100.9 (3)	N2^i^—C10—C11	118.3 (5)
C6—S2—Ag1	103.1 (2)	C7—C10—C11	121.1 (4)
C5—S2—Ag1	104.98 (18)	C10—C11—S4	116.4 (4)
C9—S3—C8	100.2 (3)	C10—C11—H11A	108.2
C11—S4—C12	100.8 (3)	S4—C11—H11A	108.2
C11—S4—Ag1^i^	94.24 (19)	C10—C11—H11B	108.2
C12—S4—Ag1^i^	103.6 (3)	S4—C11—H11B	108.2
C4—N1—C1	118.7 (5)	H11A—C11—H11B	107.3
C7—N2—C10^i^	118.7 (5)	C7—C8—S3	114.8 (4)
C7—N2—Ag1	124.7 (3)	C7—C8—H8A	108.6
C10^i^—N2—Ag1	116.1 (3)	S3—C8—H8A	108.6
N1—C1—C4^ii^	120.5 (5)	C7—C8—H8B	108.6
N1—C1—C2	116.1 (5)	S3—C8—H8B	108.6
C4^ii^—C1—C2	123.4 (5)	H8A—C8—H8B	107.6
C1—C2—S1	111.8 (4)	S3—C9—H9A	109.5
C1—C2—H2A	109.3	S3—C9—H9B	109.5
S1—C2—H2A	109.3	H9A—C9—H9B	109.5
C1—C2—H2B	109.3	S3—C9—H9C	109.5
S1—C2—H2B	109.3	H9A—C9—H9C	109.5
H2A—C2—H2B	107.9	H9B—C9—H9C	109.5
S1—C3—H3A	109.5	S4—C12—H12A	109.5
S1—C3—H3B	109.5	S4—C12—H12B	109.5
H3A—C3—H3B	109.5	H12A—C12—H12B	109.5
S1—C3—H3C	109.5	S4—C12—H12C	109.5
H3A—C3—H3C	109.5	H12A—C12—H12C	109.5
H3B—C3—H3C	109.5	H12B—C12—H12C	109.5
N1—C4—C1^ii^	120.8 (5)	O12A—N10—O11	130.3 (10)
N1—C4—C5	114.9 (5)	O11—N10—O13B	134.4 (9)
C1^ii^—C4—C5	124.3 (5)	O12A—N10—O13A	121.9 (11)
C4—C5—S2	112.6 (4)	O11—N10—O13A	106.9 (8)
C4—C5—H5A	109.1	O11—N10—O12B	108.2 (8)
S2—C5—H5A	109.1	O13B—N10—O12B	116.2 (10)
			
C4—N1—C1—C4^ii^	1.9 (8)	C10^i^—N2—C7—C8	178.5 (5)
C4—N1—C1—C2	−177.3 (5)	Ag1—N2—C7—C8	7.0 (6)
N1—C1—C2—S1	9.1 (6)	N2—C7—C10—N2^i^	1.3 (8)
C4^ii^—C1—C2—S1	−170.1 (4)	C8—C7—C10—N2^i^	−178.4 (5)
C3—S1—C2—C1	159.3 (4)	N2—C7—C10—C11	177.5 (5)
Ag1—S1—C2—C1	34.5 (4)	C8—C7—C10—C11	−2.3 (8)
C1—N1—C4—C1^ii^	−1.9 (8)	N2^i^—C10—C11—S4	−41.6 (6)
C1—N1—C4—C5	179.2 (5)	C7—C10—C11—S4	142.1 (4)
N1—C4—C5—S2	−58.1 (6)	C12—S4—C11—C10	−60.4 (5)
C1^ii^—C4—C5—S2	123.1 (5)	Ag1^i^—S4—C11—C10	44.3 (4)
C6—S2—C5—C4	−74.8 (5)	N2—C7—C8—S3	107.7 (5)
Ag1—S2—C5—C4	32.1 (4)	C10—C7—C8—S3	−72.5 (6)
C10^i^—N2—C7—C10	−1.3 (8)	C9—S3—C8—C7	−63.6 (5)
Ag1—N2—C7—C10	−172.8 (4)		

Symmetry codes: (i) −*x*, −*y*+1, −*z*+1; (ii) −*x*+1, −*y*+1, −*z*+2.

### (I) catena-Poly[[silver(I)-µ-2,3,5,6-tetrakis[(methylsulfanyl)methyl]pyrazine] nitrate] Hydrogen-bond geometry (Å, º)

**Table:**

*D*—H···*A*	*D*—H	H···*A*	*D*···*A*	*D*—H···*A*
C9—H9*C*···S2	0.96	2.86	3.650 (8)	141
C11—H11*A*···S3	0.97	2.74	3.502 (6)	136
C11—H11*B*···O13*A*	0.97	2.52	3.438 (17)	157
C2—H2*A*···O11^iii^	0.97	2.55	3.460 (9)	156
C2—H2*B*···O12*A*^iv^	0.97	2.53	3.431 (15)	154
C3—H3*C*···O12*A*^iv^	0.96	2.37	3.171 (17)	141
C3—H3*C*···O12*B*^iv^	0.96	2.57	3.364 (16)	140
C6—H6*A*···O13*A*^i^	0.96	2.52	3.375 (19)	149
C9—H9*A*···O11^v^	0.96	2.58	3.503 (10)	162

Symmetry codes: (i) −*x*, −*y*+1, −*z*+1; (iii) *x*+1/2, −*y*+1/2, *z*+1/2; (iv) −*x*+1, −*y*+1, −*z*+1; (v) *x*−1/2, −*y*+1/2, *z*+1/2.

### (II) Poly[di-µ-nitrato-bis{µ-2,3,5,6-tetrakis[(phenylsulfanyl)methyl]pyrazine}disilver] Crystal data

**Table:**

[Ag_2_(NO_3_)_2_(C_32_H_28_N_2_S_4_)]	*F*(000) = 908
*M**_r_* = 908.56	*D*_x_ = 1.762 Mg m^−^^3^
Monoclinic, *P*2_1_/*c*	Mo *K*α radiation, λ = 0.71073 Å
*a* = 11.8437 (14) Å	Cell parameters from 24 reflections
*b* = 18.5674 (14) Å	θ = 11.2–17.7°
*c* = 7.8444 (12) Å	µ = 1.44 mm^−^^1^
β = 96.856 (11)°	*T* = 293 K
*V* = 1712.7 (4) Å^3^	Block, pale yellow
*Z* = 2	0.46 × 0.46 × 0.38 mm

### (II) Poly[di-µ-nitrato-bis{µ-2,3,5,6-tetrakis[(phenylsulfanyl)methyl]pyrazine}disilver] Data collection

**Table:**

Stoe AED2 4-circle diffractometer	*R*_int_ = 0.0
Radiation source: fine-focus sealed tube	θ_max_ = 25.5°, θ_min_ = 2.1°
Graphite monochromator	*h* = −9→9
ω/2θ scans	*k* = 0→22
3178 measured reflections	*l* = 0→14
3178 independent reflections	2 standard reflections every 120 min
2606 reflections with *I* > 2σ(*I*)	intensity decay: 2%

### (II) Poly[di-µ-nitrato-bis{µ-2,3,5,6-tetrakis[(phenylsulfanyl)methyl]pyrazine}disilver] Refinement

**Table:**

Refinement on *F*^2^	Secondary atom site location: difference Fourier map
Least-squares matrix: full	Hydrogen site location: inferred from neighbouring sites
*R*[*F*^2^ > 2σ(*F*^2^)] = 0.045	H-atom parameters constrained
*wR*(*F*^2^) = 0.100	*w* = 1/[σ^2^(*F*_o_^2^) + (0.0308*P*)^2^ + 3.2342*P*] where *P* = (*F*_o_^2^ + 2*F*_c_^2^)/3
*S* = 1.16	(Δ/σ)_max_ = 0.001
3178 reflections	Δρ_max_ = 0.62 e Å^−^^3^
218 parameters	Δρ_min_ = −0.61 e Å^−^^3^
0 restraints	Extinction correction: SHELXL, Fc^*^=kFc[1+0.001xFc^2^λ^3^/sin(2θ)]^-1/4^
Primary atom site location: structure-invariant direct methods	Extinction coefficient: 0.0018 (4)

### (II) Poly[di-µ-nitrato-bis{µ-2,3,5,6-tetrakis[(phenylsulfanyl)methyl]pyrazine}disilver] Special details

**Table:**

Geometry. All esds (except the esd in the dihedral angle between two l.s. planes) are estimated using the full covariance matrix. The cell esds are taken into account individually in the estimation of esds in distances, angles and torsion angles; correlations between esds in cell parameters are only used when they are defined by crystal symmetry. An approximate (isotropic) treatment of cell esds is used for estimating esds involving l.s. planes.

### (II) Poly[di-µ-nitrato-bis{µ-2,3,5,6-tetrakis[(phenylsulfanyl)methyl]pyrazine}disilver] Fractional atomic coordinates and isotropic or equivalent isotropic displacement parameters (Å^2^)

**Table:**

	*x*	*y*	*z*	*U*_iso_*/*U*_eq_	
Ag1	1.03726 (4)	0.81386 (2)	0.31389 (6)	0.05335 (17)	
S1	0.82101 (11)	0.85133 (7)	0.23374 (16)	0.0473 (3)	
S2	0.75690 (11)	1.14870 (6)	0.53250 (17)	0.0458 (3)	
O1	1.0997 (4)	0.6845 (2)	0.2690 (5)	0.0699 (12)	
O2	1.0189 (4)	0.7981 (2)	−0.0056 (5)	0.0671 (11)	
O3	1.0734 (4)	0.5953 (2)	0.4353 (5)	0.0786 (13)	
N1	1.0335 (3)	0.94469 (19)	0.4019 (4)	0.0368 (8)	
N10	1.0647 (4)	0.6592 (2)	0.4005 (5)	0.0464 (10)	
C1	0.9323 (4)	0.9750 (2)	0.3633 (5)	0.0354 (10)	
C2	0.8967 (4)	1.0317 (2)	0.4638 (5)	0.0365 (10)	
C3	0.8593 (4)	0.9458 (2)	0.2087 (6)	0.0435 (11)	
H3A	0.8998	0.9505	0.1089	0.052*	
H3B	0.7904	0.9743	0.1886	0.052*	
C4	0.7519 (4)	0.8528 (3)	0.4246 (6)	0.0446 (11)	
C5	0.8133 (5)	0.8402 (3)	0.5828 (7)	0.0529 (13)	
H5	0.8919	0.8349	0.5915	0.063*	
C6	0.7584 (6)	0.8354 (3)	0.7273 (7)	0.0625 (15)	
H6	0.8002	0.8266	0.8332	0.075*	
C7	0.6437 (6)	0.8435 (4)	0.7171 (9)	0.0738 (18)	
H7	0.6072	0.8402	0.8154	0.089*	
C8	0.5828 (6)	0.8564 (5)	0.5616 (10)	0.091 (2)	
H8	0.5044	0.8621	0.5545	0.110*	
C9	0.6358 (5)	0.8613 (4)	0.4139 (9)	0.0767 (19)	
H9	0.5933	0.8701	0.3084	0.092*	
C10	0.7834 (4)	1.0681 (2)	0.4170 (6)	0.0406 (10)	
H10A	0.7241	1.0337	0.4334	0.049*	
H10B	0.7763	1.0797	0.2957	0.049*	
C11	0.7013 (4)	1.1194 (3)	0.7201 (6)	0.0439 (11)	
C12	0.6681 (5)	1.1731 (3)	0.8251 (7)	0.0588 (14)	
H12	0.6784	1.2211	0.7969	0.071*	
C13	0.6194 (5)	1.1561 (4)	0.9719 (8)	0.0700 (17)	
H13	0.5977	1.1926	1.0422	0.084*	
C14	0.6031 (5)	1.0855 (4)	1.0141 (7)	0.0710 (19)	
H14	0.5694	1.0742	1.1119	0.085*	
C15	0.6368 (5)	1.0312 (4)	0.9113 (7)	0.0646 (16)	
H15	0.6262	0.9832	0.9401	0.078*	
C16	0.6866 (5)	1.0481 (3)	0.7647 (7)	0.0539 (13)	
H16	0.7101	1.0115	0.6963	0.065*	

### (II) Poly[di-µ-nitrato-bis{µ-2,3,5,6-tetrakis[(phenylsulfanyl)methyl]pyrazine}disilver] Atomic displacement parameters (Å^2^)

**Table:**

	*U*^11^	*U*^22^	*U*^33^	*U*^12^	*U*^13^	*U*^23^
Ag1	0.0616 (3)	0.0377 (2)	0.0624 (3)	0.00179 (19)	0.01433 (19)	−0.00855 (18)
S1	0.0601 (8)	0.0426 (7)	0.0388 (6)	−0.0055 (6)	0.0047 (6)	−0.0088 (5)
S2	0.0516 (7)	0.0354 (6)	0.0513 (7)	0.0076 (5)	0.0109 (6)	0.0029 (5)
O1	0.107 (3)	0.057 (2)	0.052 (2)	0.015 (2)	0.034 (2)	0.0080 (19)
O2	0.097 (3)	0.057 (2)	0.052 (2)	−0.019 (2)	0.029 (2)	−0.0007 (18)
O3	0.130 (4)	0.040 (2)	0.070 (3)	0.010 (2)	0.024 (3)	0.0085 (19)
N1	0.047 (2)	0.0323 (19)	0.0303 (19)	0.0022 (17)	0.0034 (16)	−0.0011 (15)
N10	0.058 (3)	0.041 (2)	0.041 (2)	0.003 (2)	0.0092 (19)	0.0019 (19)
C1	0.047 (3)	0.028 (2)	0.031 (2)	−0.0015 (19)	0.0042 (19)	0.0014 (17)
C2	0.047 (3)	0.031 (2)	0.032 (2)	−0.003 (2)	0.006 (2)	0.0035 (18)
C3	0.059 (3)	0.037 (2)	0.032 (2)	0.003 (2)	−0.003 (2)	0.0010 (19)
C4	0.050 (3)	0.040 (3)	0.044 (3)	−0.003 (2)	0.005 (2)	−0.002 (2)
C5	0.046 (3)	0.062 (3)	0.052 (3)	0.009 (3)	0.009 (2)	0.006 (3)
C6	0.078 (4)	0.064 (4)	0.048 (3)	0.011 (3)	0.015 (3)	0.006 (3)
C7	0.079 (5)	0.080 (4)	0.069 (4)	−0.001 (4)	0.035 (4)	−0.002 (3)
C8	0.049 (4)	0.132 (7)	0.096 (6)	−0.002 (4)	0.022 (4)	−0.005 (5)
C9	0.054 (4)	0.109 (6)	0.065 (4)	−0.003 (4)	−0.005 (3)	−0.004 (4)
C10	0.048 (3)	0.036 (2)	0.037 (2)	0.002 (2)	0.004 (2)	0.000 (2)
C11	0.040 (3)	0.050 (3)	0.043 (3)	−0.001 (2)	0.006 (2)	−0.003 (2)
C12	0.065 (4)	0.056 (3)	0.057 (3)	0.002 (3)	0.013 (3)	−0.017 (3)
C13	0.066 (4)	0.090 (5)	0.055 (4)	0.002 (4)	0.011 (3)	−0.019 (3)
C14	0.052 (3)	0.122 (6)	0.041 (3)	−0.012 (4)	0.010 (3)	0.001 (3)
C15	0.060 (4)	0.079 (4)	0.055 (3)	−0.016 (3)	0.009 (3)	0.012 (3)
C16	0.060 (3)	0.052 (3)	0.051 (3)	−0.002 (3)	0.013 (3)	−0.001 (2)

### (II) Poly[di-µ-nitrato-bis{µ-2,3,5,6-tetrakis[(phenylsulfanyl)methyl]pyrazine}disilver] Geometric parameters (Å, º)

**Table:**

Ag1—N1	2.527 (4)	C4—C9	1.376 (8)
Ag1—S1	2.6560 (15)	C4—C5	1.382 (7)
Ag1—S2^i^	2.6790 (14)	C5—C6	1.376 (7)
Ag1—O1	2.551 (4)	C5—H5	0.9300
Ag1—O2	2.507 (4)	C6—C7	1.359 (9)
Ag1—O2^ii^	2.539 (4)	C6—H6	0.9300
S1—C4	1.790 (5)	C7—C8	1.363 (10)
S1—C3	1.828 (5)	C7—H7	0.9300
S2—C11	1.768 (5)	C8—C9	1.385 (9)
S2—C10	1.796 (4)	C8—H8	0.9300
S2—Ag1^i^	2.6790 (14)	C9—H9	0.9300
O1—N10	1.248 (5)	C10—H10A	0.9700
O2—N10^iii^	1.250 (5)	C10—H10B	0.9700
O2—Ag1^iii^	2.539 (4)	C11—C12	1.381 (7)
O3—N10	1.219 (5)	C11—C16	1.385 (7)
N1—C1	1.326 (6)	C12—C13	1.385 (8)
N1—C2^i^	1.334 (5)	C12—H12	0.9300
N10—O2^ii^	1.250 (5)	C13—C14	1.372 (9)
C1—C2	1.409 (6)	C13—H13	0.9300
C1—C3	1.504 (6)	C14—C15	1.380 (9)
C2—N1^i^	1.334 (5)	C14—H14	0.9300
C2—C10	1.508 (6)	C15—C16	1.390 (7)
C3—H3A	0.9700	C15—H15	0.9300
C3—H3B	0.9700	C16—H16	0.9300
			
N1—Ag1—S1	76.40 (9)	C9—C4—C5	119.3 (5)
N1—Ag1—S2^i^	70.89 (9)	C9—C4—S1	120.3 (4)
S1—Ag1—S2^i^	146.98 (4)	C5—C4—S1	120.2 (4)
O2—Ag1—N1	112.54 (12)	C6—C5—C4	120.1 (5)
O2—Ag1—O2^ii^	117.32 (8)	C6—C5—H5	120.0
N1—Ag1—O2^ii^	128.98 (12)	C4—C5—H5	120.0
O2—Ag1—O1	75.15 (13)	C7—C6—C5	120.8 (6)
N1—Ag1—O1	163.34 (14)	C7—C6—H6	119.6
O2^ii^—Ag1—O1	49.56 (12)	C5—C6—H6	119.6
O2—Ag1—S1	80.10 (11)	C6—C7—C8	119.4 (6)
O2^ii^—Ag1—S1	101.67 (11)	C6—C7—H7	120.3
O1—Ag1—S1	120.09 (11)	C8—C7—H7	120.3
O2—Ag1—S2^i^	116.46 (10)	C7—C8—C9	121.1 (6)
O2^ii^—Ag1—S2^i^	95.47 (11)	C7—C8—H8	119.5
O1—Ag1—S2^i^	92.47 (11)	C9—C8—H8	119.5
C4—S1—C3	102.6 (2)	C4—C9—C8	119.4 (6)
C4—S1—Ag1	109.31 (17)	C4—C9—H9	120.3
C3—S1—Ag1	91.85 (17)	C8—C9—H9	120.3
C11—S2—C10	105.6 (2)	C2—C10—S2	117.1 (3)
C11—S2—Ag1^i^	96.58 (16)	C2—C10—H10A	108.0
C10—S2—Ag1^i^	103.79 (16)	S2—C10—H10A	108.0
N10—O1—Ag1	96.3 (3)	C2—C10—H10B	108.0
N10^iii^—O2—Ag1	121.5 (3)	S2—C10—H10B	108.0
N10^iii^—O2—Ag1^iii^	96.8 (3)	H10A—C10—H10B	107.3
Ag1—O2—Ag1^iii^	130.58 (17)	C12—C11—C16	119.1 (5)
C1—N1—C2^i^	119.9 (4)	C12—C11—S2	115.8 (4)
C1—N1—Ag1	113.0 (3)	C16—C11—S2	125.1 (4)
C2^i^—N1—Ag1	120.2 (3)	C11—C12—C13	120.5 (6)
O3—N10—O1	121.6 (4)	C11—C12—H12	119.8
O3—N10—O2^ii^	121.1 (4)	C13—C12—H12	119.8
O1—N10—O2^ii^	117.3 (4)	C14—C13—C12	120.3 (6)
N1—C1—C2	120.8 (4)	C14—C13—H13	119.9
N1—C1—C3	116.6 (4)	C12—C13—H13	119.9
C2—C1—C3	122.6 (4)	C13—C14—C15	119.9 (5)
N1^i^—C2—C1	119.2 (4)	C13—C14—H14	120.0
N1^i^—C2—C10	119.6 (4)	C15—C14—H14	120.0
C1—C2—C10	121.1 (4)	C14—C15—C16	119.9 (6)
C1—C3—S1	112.7 (3)	C14—C15—H15	120.0
C1—C3—H3A	109.1	C16—C15—H15	120.0
S1—C3—H3A	109.1	C11—C16—C15	120.3 (5)
C1—C3—H3B	109.1	C11—C16—H16	119.8
S1—C3—H3B	109.1	C15—C16—H16	119.8
H3A—C3—H3B	107.8		
			
Ag1—O1—N10—O3	178.4 (5)	C5—C6—C7—C8	0.0 (10)
Ag1—O1—N10—O2^ii^	−0.7 (5)	C6—C7—C8—C9	0.2 (12)
C2^i^—N1—C1—C2	−0.7 (7)	C5—C4—C9—C8	−0.4 (10)
Ag1—N1—C1—C2	150.3 (3)	S1—C4—C9—C8	174.8 (6)
C2^i^—N1—C1—C3	179.3 (4)	C7—C8—C9—C4	0.0 (12)
Ag1—N1—C1—C3	−29.7 (5)	N1^i^—C2—C10—S2	7.1 (6)
N1—C1—C2—N1^i^	0.7 (7)	C1—C2—C10—S2	−170.1 (3)
C3—C1—C2—N1^i^	−179.3 (4)	C11—S2—C10—C2	−86.4 (4)
N1—C1—C2—C10	177.9 (4)	Ag1^i^—S2—C10—C2	14.6 (4)
C3—C1—C2—C10	−2.2 (6)	C10—S2—C11—C12	−176.3 (4)
N1—C1—C3—S1	62.9 (5)	Ag1^i^—S2—C11—C12	77.3 (4)
C2—C1—C3—S1	−117.1 (4)	C10—S2—C11—C16	1.8 (5)
C4—S1—C3—C1	57.2 (4)	Ag1^i^—S2—C11—C16	−104.5 (5)
Ag1—S1—C3—C1	−53.1 (3)	C16—C11—C12—C13	−0.7 (8)
C3—S1—C4—C9	92.2 (5)	S2—C11—C12—C13	177.6 (5)
Ag1—S1—C4—C9	−171.3 (5)	C11—C12—C13—C14	−0.4 (9)
C3—S1—C4—C5	−92.6 (5)	C12—C13—C14—C15	1.0 (10)
Ag1—S1—C4—C5	3.9 (5)	C13—C14—C15—C16	−0.4 (9)
C9—C4—C5—C6	0.6 (9)	C12—C11—C16—C15	1.3 (8)
S1—C4—C5—C6	−174.6 (4)	S2—C11—C16—C15	−176.8 (4)
C4—C5—C6—C7	−0.4 (9)	C14—C15—C16—C11	−0.8 (9)

Symmetry codes: (i) −*x*+2, −*y*+2, −*z*+1; (ii) *x*, −*y*+3/2, *z*+1/2; (iii) *x*, −*y*+3/2, *z*−1/2.

### (III) Poly[[trinitrato{µ6-2,3,5,6-tetrakis[(pyridin-2-ylsulfanyl)methyl]pyrazine}trisilver(I)] Crystal data

**Table:**

[Ag_3_(NO_3_)_3_(C_28_H_24_N_6_S_4_)]	*F*(000) = 2128
*M**_r_* = 1082.41	*D*_x_ = 2.082 Mg m^−^^3^
Monoclinic, *C*2/*c*	Mo *K*α radiation, λ = 0.71073 Å
*a* = 13.6319 (9) Å	Cell parameters from 5000 reflections
*b* = 16.2211 (10) Å	θ = 2.0–25.9°
*c* = 15.7201 (11) Å	µ = 1.99 mm^−^^1^
β = 96.607 (8)°	*T* = 223 K
*V* = 3453.0 (4) Å^3^	Needle, pale yellow
*Z* = 4	0.45 × 0.08 × 0.08 mm

### (III) Poly[[trinitrato{µ6-2,3,5,6-tetrakis[(pyridin-2-ylsulfanyl)methyl]pyrazine}trisilver(I)] Data collection

**Table:**

STOE IPDS 1 diffractometer	3311 independent reflections
Radiation source: fine-focus sealed tube	1936 reflections with *I* > 2σ(*I*)
Plane graphite monochromator	*R*_int_ = 0.072
φ rotation scans	θ_max_ = 25.9°, θ_min_ = 2.0°
Absorption correction: multi-scan (MULABS; Spek, 2009)	*h* = −16→16
*T*_min_ = 0.949, *T*_max_ = 1.000	*k* = −19→19
13264 measured reflections	*l* = −19→18

### (III) Poly[[trinitrato{µ6-2,3,5,6-tetrakis[(pyridin-2-ylsulfanyl)methyl]pyrazine}trisilver(I)] Refinement

**Table:**

Refinement on *F*^2^	Secondary atom site location: difference Fourier map
Least-squares matrix: full	Hydrogen site location: inferred from neighbouring sites
*R*[*F*^2^ > 2σ(*F*^2^)] = 0.030	H-atom parameters constrained
*wR*(*F*^2^) = 0.052	*w* = 1/[σ^2^(*F*_o_^2^) + (0.0179*P*)^2^] where *P* = (*F*_o_^2^ + 2*F*_c_^2^)/3
*S* = 0.76	(Δ/σ)_max_ < 0.001
3311 reflections	Δρ_max_ = 0.43 e Å^−^^3^
242 parameters	Δρ_min_ = −0.44 e Å^−^^3^
0 restraints	Extinction correction: SHELXL, Fc^*^=kFc[1+0.001xFc^2^λ^3^/sin(2θ)]^-1/4^
Primary atom site location: structure-invariant direct methods	Extinction coefficient: 0.00014 (3)

### (III) Poly[[trinitrato{µ6-2,3,5,6-tetrakis[(pyridin-2-ylsulfanyl)methyl]pyrazine}trisilver(I)] Special details

**Table:**

Geometry. All esds (except the esd in the dihedral angle between two l.s. planes) are estimated using the full covariance matrix. The cell esds are taken into account individually in the estimation of esds in distances, angles and torsion angles; correlations between esds in cell parameters are only used when they are defined by crystal symmetry. An approximate (isotropic) treatment of cell esds is used for estimating esds involving l.s. planes.

### (III) Poly[[trinitrato{µ6-2,3,5,6-tetrakis[(pyridin-2-ylsulfanyl)methyl]pyrazine}trisilver(I)] Fractional atomic coordinates and isotropic or equivalent isotropic displacement parameters (Å^2^)

**Table:**

	*x*	*y*	*z*	*U*_iso_*/*U*_eq_	
Ag1	0.01942 (2)	0.37808 (2)	−0.08024 (3)	0.04096 (12)	
Ag2	0.5000	−0.03325 (3)	0.2500	0.05015 (18)	
S1	0.09150 (7)	0.43818 (6)	0.08153 (8)	0.0309 (3)	
S2	0.37324 (7)	0.12904 (6)	0.20654 (7)	0.0293 (3)	
O11	0.0033 (3)	0.2151 (3)	−0.1140 (3)	0.0993 (17)	
O12	0.0505 (3)	0.1241 (2)	−0.0186 (3)	0.0881 (13)	
O13	−0.0038 (3)	0.2406 (3)	0.0181 (3)	0.0871 (14)	
O21	0.5000	−0.1915 (3)	0.2500	0.082 (2)	
O22	0.5755 (3)	−0.3025 (3)	0.2419 (3)	0.0890 (15)	
N1	0.1890 (2)	0.31443 (19)	−0.0280 (2)	0.0228 (8)	
N2	0.1418 (2)	0.59202 (19)	0.0729 (2)	0.0278 (8)	
N3	0.3466 (2)	−0.0278 (2)	0.1875 (2)	0.0277 (8)	
N11	0.0173 (3)	0.1915 (3)	−0.0375 (4)	0.0643 (14)	
N21	0.5000	−0.2669 (3)	0.2500	0.0365 (13)	
C1	0.2093 (3)	0.2984 (2)	0.0553 (3)	0.0232 (9)	
C2	0.2715 (3)	0.2341 (2)	0.0833 (3)	0.0217 (9)	
C3	0.1631 (3)	0.3497 (2)	0.1204 (3)	0.0306 (11)	
H3A	0.2161	0.3689	0.1632	0.037*	
H3B	0.1204	0.3136	0.1499	0.037*	
C4	0.1816 (3)	0.5165 (2)	0.0793 (3)	0.0295 (10)	
C5	0.2821 (3)	0.5034 (3)	0.0844 (3)	0.0517 (14)	
H5	0.3082	0.4497	0.0867	0.062*	
C6	0.3433 (3)	0.5714 (3)	0.0861 (4)	0.0617 (16)	
H6	0.4122	0.5645	0.0912	0.074*	
C7	0.3033 (3)	0.6488 (3)	0.0803 (3)	0.0518 (14)	
H7	0.3442	0.6956	0.0814	0.062*	
C8	0.2039 (3)	0.6569 (3)	0.0731 (3)	0.0407 (12)	
H8	0.1768	0.7102	0.0679	0.049*	
C9	0.2962 (3)	0.2171 (2)	0.1786 (3)	0.0279 (10)	
H9A	0.2341	0.2099	0.2036	0.034*	
H9B	0.3290	0.2660	0.2053	0.034*	
C11	0.2968 (3)	−0.0982 (3)	0.1679 (3)	0.0406 (12)	
H11	0.3317	−0.1483	0.1722	0.049*	
C12	0.1982 (4)	−0.0998 (3)	0.1421 (3)	0.0568 (15)	
H12	0.1653	−0.1501	0.1296	0.068*	
C13	0.1476 (4)	−0.0263 (3)	0.1347 (4)	0.0654 (17)	
H13	0.0792	−0.0260	0.1174	0.079*	
C14	0.1966 (3)	0.0468 (3)	0.1524 (3)	0.0492 (13)	
H14	0.1631	0.0976	0.1466	0.059*	
C15	0.2959 (3)	0.0433 (2)	0.1789 (3)	0.0276 (10)	

### (III) Poly[[trinitrato{µ6-2,3,5,6-tetrakis[(pyridin-2-ylsulfanyl)methyl]pyrazine}trisilver(I)] Atomic displacement parameters (Å^2^)

**Table:**

	*U*^11^	*U*^22^	*U*^33^	*U*^12^	*U*^13^	*U*^23^
Ag1	0.02369 (17)	0.0330 (2)	0.0673 (3)	0.00667 (16)	0.01013 (16)	0.0075 (2)
Ag2	0.0223 (3)	0.0276 (3)	0.0975 (5)	0.000	−0.0062 (3)	0.000
S1	0.0246 (5)	0.0246 (6)	0.0447 (7)	0.0066 (5)	0.0092 (5)	0.0023 (5)
S2	0.0227 (5)	0.0279 (6)	0.0354 (6)	0.0041 (5)	−0.0043 (5)	0.0031 (6)
O11	0.080 (3)	0.112 (4)	0.097 (4)	−0.053 (3)	−0.026 (3)	0.032 (3)
O12	0.058 (2)	0.044 (2)	0.160 (4)	0.010 (2)	0.000 (2)	0.016 (3)
O13	0.053 (2)	0.061 (3)	0.154 (4)	−0.013 (2)	0.037 (3)	−0.001 (3)
O21	0.106 (4)	0.025 (3)	0.129 (5)	0.000	0.077 (4)	0.000
O22	0.070 (3)	0.100 (3)	0.105 (3)	0.054 (3)	0.041 (3)	0.036 (3)
N1	0.0195 (17)	0.0221 (18)	0.027 (2)	0.0040 (14)	0.0037 (16)	0.0043 (16)
N2	0.0240 (18)	0.0266 (19)	0.033 (2)	0.0020 (15)	0.0058 (16)	−0.0011 (16)
N3	0.0243 (18)	0.0279 (19)	0.030 (2)	−0.0017 (16)	0.0012 (16)	0.0009 (18)
N11	0.030 (2)	0.048 (3)	0.114 (5)	−0.015 (2)	0.004 (3)	−0.004 (4)
N21	0.032 (3)	0.037 (3)	0.041 (4)	0.000	0.005 (3)	0.000
C1	0.017 (2)	0.018 (2)	0.034 (3)	0.0004 (16)	0.0019 (19)	0.000 (2)
C2	0.017 (2)	0.018 (2)	0.030 (3)	0.0008 (16)	0.0046 (19)	0.005 (2)
C3	0.034 (2)	0.029 (2)	0.031 (3)	0.0115 (19)	0.016 (2)	0.006 (2)
C4	0.022 (2)	0.035 (3)	0.031 (3)	0.0032 (19)	0.0019 (19)	0.000 (2)
C5	0.033 (3)	0.043 (3)	0.082 (4)	0.010 (2)	0.014 (3)	−0.001 (3)
C6	0.026 (3)	0.062 (4)	0.098 (5)	−0.002 (3)	0.012 (3)	−0.002 (3)
C7	0.036 (3)	0.050 (3)	0.070 (4)	−0.010 (2)	0.008 (3)	−0.009 (3)
C8	0.037 (3)	0.034 (3)	0.053 (3)	0.001 (2)	0.010 (2)	0.000 (2)
C9	0.026 (2)	0.023 (2)	0.034 (3)	0.0067 (18)	0.000 (2)	0.000 (2)
C11	0.041 (3)	0.032 (3)	0.048 (3)	−0.005 (2)	−0.003 (2)	0.003 (2)
C12	0.048 (3)	0.044 (3)	0.072 (4)	−0.015 (3)	−0.020 (3)	0.006 (3)
C13	0.034 (3)	0.054 (4)	0.100 (5)	−0.007 (3)	−0.026 (3)	0.018 (3)
C14	0.032 (3)	0.038 (3)	0.074 (4)	0.006 (2)	−0.010 (2)	0.014 (3)
C15	0.025 (2)	0.030 (2)	0.028 (3)	0.0002 (19)	0.0030 (19)	0.006 (2)

### (III) Poly[[trinitrato{µ6-2,3,5,6-tetrakis[(pyridin-2-ylsulfanyl)methyl]pyrazine}trisilver(I)] Geometric parameters (Å, º)

**Table:**

Ag1—N1	2.578 (3)	N21—O22^iii^	1.200 (4)
Ag1—N2^i^	2.267 (3)	C1—C2	1.384 (5)
Ag1—S1	2.7943 (13)	C1—C3	1.512 (5)
Ag1—S2^ii^	2.6010 (11)	C2—N1^ii^	1.331 (5)
Ag1—O11	2.700 (5)	C2—C9	1.523 (5)
Ag1—O13	2.752 (5)	C3—H3A	0.9800
Ag2—N3	2.208 (3)	C3—H3B	0.9800
Ag2—N3^iii^	2.208 (3)	C4—C5	1.379 (6)
Ag2—O21	2.567 (5)	C5—C6	1.382 (6)
S1—C4	1.770 (4)	C5—H5	0.9400
S1—C3	1.802 (4)	C6—C7	1.368 (6)
S2—C15	1.769 (4)	C6—H6	0.9400
S2—C9	1.798 (4)	C7—C8	1.353 (6)
S2—Ag1^ii^	2.6011 (11)	C7—H7	0.9400
O11—N11	1.255 (6)	C8—H8	0.9400
O12—N11	1.208 (5)	C9—H9A	0.9800
O13—N11	1.239 (6)	C9—H9B	0.9800
O21—N21	1.223 (6)	C11—C12	1.360 (6)
O22—N21	1.200 (4)	C11—H11	0.9400
N1—C2^ii^	1.331 (5)	C12—C13	1.377 (6)
N1—C1	1.332 (5)	C12—H12	0.9400
N2—C4	1.339 (5)	C13—C14	1.374 (6)
N2—C8	1.351 (5)	C13—H13	0.9400
N2—Ag1^i^	2.266 (3)	C14—C15	1.372 (6)
N3—C15	1.343 (5)	C14—H14	0.9400
N3—C11	1.345 (5)		
			
N1—Ag1—N2^i^	155.31 (11)	N1^ii^—C2—C9	118.4 (3)
S1—Ag1—S2^ii^	122.71 (3)	C1—C2—C9	120.4 (4)
S1—Ag1—N1	68.98 (7)	C1—C3—S1	117.4 (3)
S1—Ag1—N2^i^	96.92 (8)	C1—C3—H3A	107.9
S2^ii^—Ag1—N1	70.29 (7)	S1—C3—H3A	107.9
S2^ii^—Ag1—N2^i^	133.03 (8)	C1—C3—H3B	107.9
S1—Ag1—O11	122.18 (10)	S1—C3—H3B	107.9
S1—Ag1—O13	79.78 (10)	H3A—C3—H3B	107.2
S2^ii^—Ag1—O11	81.18 (10)	N2—C4—C5	122.4 (4)
S2^ii^—Ag1—O13	120.26 (10)	N2—C4—S1	112.5 (3)
O11—Ag1—N1	73.76 (11)	C5—C4—S1	125.1 (3)
O11—Ag1—N2^i^	99.33 (12)	C4—C5—C6	118.2 (4)
O13—Ag1—N1	69.73 (11)	C4—C5—H5	120.9
O13—Ag1—N2^i^	88.28 (12)	C6—C5—H5	120.9
O11—Ag1—O13	45.99 (14)	C7—C6—C5	119.7 (4)
N3—Ag2—N3^iii^	175.41 (12)	C7—C6—H6	120.1
O21—Ag2—N3	92.30 (9)	C5—C6—H6	120.1
O21—Ag2—N3^iii^	92.30 (9)	C8—C7—C6	118.8 (4)
C4—S1—C3	103.19 (19)	C8—C7—H7	120.6
C4—S1—Ag1	113.84 (14)	C6—C7—H7	120.6
C3—S1—Ag1	98.68 (14)	N2—C8—C7	123.1 (4)
C15—S2—C9	104.50 (18)	N2—C8—H8	118.4
C15—S2—Ag1^ii^	98.56 (13)	C7—C8—H8	118.4
C9—S2—Ag1^ii^	102.31 (13)	C2—C9—S2	116.1 (3)
N21—O21—Ag2	180.0	C2—C9—H9A	108.3
C2^ii^—N1—C1	118.2 (3)	S2—C9—H9A	108.3
C2^ii^—N1—Ag1	116.4 (3)	C2—C9—H9B	108.3
C1—N1—Ag1	118.0 (2)	S2—C9—H9B	108.3
C4—N2—C8	117.6 (3)	H9A—C9—H9B	107.4
C4—N2—Ag1^i^	125.4 (2)	N3—C11—C12	122.7 (4)
C8—N2—Ag1^i^	116.4 (3)	N3—C11—H11	118.6
C15—N3—C11	117.8 (3)	C12—C11—H11	118.6
C15—N3—Ag2	121.9 (3)	C11—C12—C13	118.5 (4)
C11—N3—Ag2	119.7 (3)	C11—C12—H12	120.8
O12—N11—O13	121.3 (6)	C13—C12—H12	120.8
O12—N11—O11	121.5 (6)	C14—C13—C12	120.3 (4)
O13—N11—O11	117.2 (6)	C14—C13—H13	119.9
O22—N21—O22^iii^	122.4 (6)	C12—C13—H13	119.9
O22—N21—O21	118.8 (3)	C15—C14—C13	117.7 (4)
O22^iii^—N21—O21	118.8 (3)	C15—C14—H14	121.1
N1—C1—C2	120.7 (3)	C13—C14—H14	121.1
N1—C1—C3	120.1 (3)	N3—C15—C14	123.0 (4)
C2—C1—C3	119.2 (4)	N3—C15—S2	111.5 (3)
N1^ii^—C2—C1	121.1 (4)	C14—C15—S2	125.5 (3)
			
C2^ii^—N1—C1—C2	1.2 (6)	C5—C6—C7—C8	0.0 (8)
Ag1—N1—C1—C2	150.0 (3)	C4—N2—C8—C7	−0.8 (6)
C2^ii^—N1—C1—C3	−177.7 (3)	Ag1^i^—N2—C8—C7	171.1 (4)
Ag1—N1—C1—C3	−28.9 (4)	C6—C7—C8—N2	1.4 (8)
N1—C1—C2—N1^ii^	−1.3 (6)	N1^ii^—C2—C9—S2	−2.7 (5)
C3—C1—C2—N1^ii^	177.7 (3)	C1—C2—C9—S2	177.3 (3)
N1—C1—C2—C9	178.7 (3)	C15—S2—C9—C2	−72.0 (3)
C3—C1—C2—C9	−2.3 (5)	Ag1^ii^—S2—C9—C2	30.3 (3)
N1—C1—C3—S1	−6.3 (5)	C15—N3—C11—C12	−1.6 (6)
C2—C1—C3—S1	174.7 (3)	Ag2—N3—C11—C12	169.3 (4)
C4—S1—C3—C1	−85.5 (3)	N3—C11—C12—C13	0.9 (7)
Ag1—S1—C3—C1	31.6 (3)	C11—C12—C13—C14	0.6 (8)
C8—N2—C4—C5	−1.0 (6)	C12—C13—C14—C15	−1.2 (8)
Ag1^i^—N2—C4—C5	−172.2 (3)	C11—N3—C15—C14	0.9 (6)
C8—N2—C4—S1	178.2 (3)	Ag2—N3—C15—C14	−169.7 (3)
Ag1^i^—N2—C4—S1	7.1 (4)	C11—N3—C15—S2	−179.5 (3)
C3—S1—C4—N2	−164.1 (3)	Ag2—N3—C15—S2	9.8 (4)
Ag1—S1—C4—N2	90.0 (3)	C13—C14—C15—N3	0.4 (7)
C3—S1—C4—C5	15.1 (5)	C13—C14—C15—S2	−179.1 (4)
Ag1—S1—C4—C5	−90.8 (4)	C9—S2—C15—N3	173.2 (3)
N2—C4—C5—C6	2.3 (7)	Ag1^ii^—S2—C15—N3	68.1 (3)
S1—C4—C5—C6	−176.8 (4)	C9—S2—C15—C14	−7.2 (4)
C4—C5—C6—C7	−1.7 (8)	Ag1^ii^—S2—C15—C14	−112.4 (4)

Symmetry codes: (i) −*x*, −*y*+1, −*z*; (ii) −*x*+1/2, −*y*+1/2, −*z*; (iii) −*x*+1, *y*, −*z*+1/2.

### (III) Poly[[trinitrato{µ6-2,3,5,6-tetrakis[(pyridin-2-ylsulfanyl)methyl]pyrazine}trisilver(I)] Hydrogen-bond geometry (Å, º)

**Table:**

*D*—H···*A*	*D*—H	H···*A*	*D*···*A*	*D*—H···*A*
C11—H11···O21	0.94	2.57	3.287 (5)	133
C3—H3*B*···O21^iv^	0.98	2.40	3.253 (4)	145
C3—H3*B*···O22^iv^	0.98	2.49	3.420 (6)	158
C7—H7···O13^v^	0.94	2.51	3.268 (6)	138
C9—H9*A*···O22^iv^	0.98	2.32	3.291 (6)	171
C12—H12···O11^vi^	0.94	2.51	3.310 (7)	142
C14—H14···O22^iv^	0.94	2.59	3.349 (7)	138

Symmetry codes: (iv) *x*−1/2, *y*+1/2, *z*; (v) *x*+1/2, *y*+1/2, *z*; (vi) −*x*, −*y*, −*z*.
